# The Consequences of Stigma for Knowledge Production: Sheep Producers' Attitudes to Footrot Diagnostics and Control in Australia

**DOI:** 10.3389/fvets.2020.00354

**Published:** 2020-06-25

**Authors:** Nickala Best, Ramón Menéndez, Grant Rawlin, Robert Suter, Brendan Rodoni, Travis Beddoe

**Affiliations:** ^1^Department of Animal, Plant and Soil Science and Centre for AgriBioscience (AgriBio), La Trobe University, Melbourne, VIC, Australia; ^2^Department of Management, Sport and Tourism, La Trobe University, Melbourne, VIC, Australia; ^3^Department of Economic Development, Jobs, Transport and Resources Centre for AgriBioscience (AgriBio), Victorian Government, Melbourne, VIC, Australia; ^4^Agriculture Services and Biosecurity Operations, Department of Economic Development, Jobs, Transport and Resources, Attwood, VIC, Australia

**Keywords:** sheep producers' attitudes, footrot, survey research, stigma, trust, sociology of knowledge

## Abstract

In Australia, there is documented confusion from producers around the clinical disease of footrot, and anecdotally, knowledge of what tools are available for the diagnosis and management of footrot. When discussing footrot with producers, the authors noted a hesitation to discuss, with denial often expressed. The disease can be debilitating, both on the sheep's welfare and the producer's well-being, as it is a very difficult disease to manage and eradicate. Gaining an understanding of producer perceptions of the disease may help ensure any future actions for management and control are in-line with those identified by producers. A combination of a web-based, and manually distributed surveys of 45 sheep producers was conducted. This included closed- and open-ended questions, multi check box, and Likert scales. Responses were quantified by descriptive statistics and a thematic analysis conducted of short answers. The results of this survey indicate satisfaction with footrot diagnostics is low, while satisfaction with control methods is high. There was also a poor general understanding of footrot as a disease, and a general distrust in peers when it comes to correct management of footrot. This research addresses a gap in the literature about how sociological conditions affect diagnosis and control of footrot disease. It provides three main recommendations—simplifying the diagnostic message, encouraging a culture of trust among sheep producers and increasing governmental support—as a way to tackle this problem.

## Introduction

Footrot has been a disease of sheep in Australia for many years, with the disease first mentioned in South Australia in 1875 (1), and some of the first published research on the disease occurring in 1941 (2). Recent studies have increased the molecular understanding of the disease (3–7), but in traditional etiology, sociological conditions where diseases are diagnosed and treated are not often explored. This research explores how sociological factors can affect knowledge-production about this disease; more concretely, how sheep producers' experience and general attitudes toward the disease contribute to the use of diagnostics and the on-farm management of the disease.

Many of the agricultural surveys that have been conducted on footrot disease are on general management practices, and not necessarily the social aspects that can come from livestock infections on farm. Some studies have shown how social factors are relevant to understand veterinary disease or environmental hazards (8–16). These studies have explored similar topics to the one explored in this paper, with similar approaches, however none of them are about footrot disease in Australia. For example, they examine farmers perceptions' and management of bovine tuberculosis (bTB) in the UK (15, 16), issues of trust in governments related to handling biosecurity risks both in Australia and the UK (9–11), or the impact of cultural scripts on farmers managing their flocks disease (8, 12, 14). Only one study to date have explored the social aspects of footrot (17). This study investigated Victorian sheep producers' attitudes to footrot in Australia, and found there was a poor understanding of diagnosis, control and eradication. This study also identified footrot as the subject of “myth and legend” and as a “social disease” in the farming community, with the owners of footrot flocks becoming isolated by the community. The results presented here show that little has changed in 20 years, emphasizing the negative impact that social stigma has on producers' diagnosis, control and eradication of this disease. However, in this paper we examine the potential causes of this stigma, its consequences, and offer three recommendations to effectively tackle footrot disease.

The effects of a social stigma associated with a disease and its impact on knowledge production have not yet been investigated. Following sociologist Merton's (18) framework of a “paradigm for sociology of knowledge,” it is not possible to separate the production of knowledge from its sociological conditions. We argue that dealing with the social level of the footrot disease we can understand why this disease is not being addressed properly and openly, which could be done by merging institutional and sheep producers' efforts.

Footrot is a complex disease. The bacterium primarily responsible for footrot, *Dichelobacter nodosus*, is genetically diverse and difficult to work with in a laboratory. Its manifestation is in accord with the environmental conditions (19), where diagnostics for footrot can identify infection with *D. nodosus*, however may not match the clinical expression of the disease. The expression of clinical footrot is variable—three factors (bacterial genetics, sheep genetics, and environmental conditions) influence the severity of disease seen and add complexity to any laboratory-based diagnostic for the disease. Because of this, debate in Australia surrounds the use of laboratory-based diagnostics that focus on the detection of the pathogen (20, 21), with these techniques not widely offered and often only available through veterinarians or government laboratories. Anecdotally, this has resulted in speculation and misinformation in industry, obscuring the understanding of the disease further for the producer, and a fear that a formal diagnosis will result in enforcement of legislation present in some states of Australia. Clinically, footrot is spoken about in terms of “virulent” and “benign,” with a scoring system of lesion severity used to decide if a flock has virulent or benign footrot. The disease covers all spectrums of clinical expression, often within the one flock. To the layman, the word virulent conjures images of being dangerous and hostile, while benign implies almost a kindly disposition.

Sheep producers' self-diagnosis of their affected flock often reflects that gray distinction embedded in the institutional discourse about the disease, which may make matters worse. They may prefer to label their affected sheep as having a benign expression of the disease—especially if symptoms are not overly severe—because this might preserve their reputation and their sales of sheep. On the contrary, a virulent diagnosis would damage their reputation and produce economic loss. While institutional knowledge—such as the one that comes from generally accepted science (e.g., medicine or veterinary)—may have a higher perceived degree of social consensus than other forms of less legitimate knowledge, in fact institutional knowledge does not mean that the process of furthering knowledge within a science is free from doubt and uncertainty (22). This is the case of footrot disease, and of veterinary practices more broadly (13).

This complexity of diagnosis comes intertwined with a strong social stigma associated with the disease among the sheep producers' community. This stigma establishes that the producer that has footrot in their flock cannot handle his or her farm well; this is something that has been reflected in related literature with other types of disease [(10), p. 369]. Due to this fear of stigmatization, those affected often deal with the disease inhouse, carrying out an often inaccurate self-diagnosis and treatment of the flock, without engaging a professional such as a veterinarian or footrot contractor, as official diagnosis can be undesirable in terms of both legislation and negative labeling in the community. This negative labeling is helped by the wording and legislation surrounding footrot. The image of being under “disease control legislation,” which is designed to protect the wider industry and improve animal welfare, still implies that an individual is being managed by the state, requiring governmental “intervention” or perhaps even that he or she has broken the law or done something wrong, rather than it being an unfortunate circumstance.

The stigma also makes producers try to “get rid” of the problem quickly by selling stock on without overtly declaring their problem or culling suspiciously affected flock. Once self-diagnosed, and if private treatment is not successful or too costly, the sheep will be sold or culled. This strategy, while it may produce a short-term economic benefit for the producer who sells—given that he or she is not caught up in selling affected flock—has disastrous consequences in the long-term for the community of sheep producers, generating a culture of distrust that contributes to foster the continuation of a closed loop of communication and misinformation, and poses a biosecurity risk. Thus, economic interests drive knowledge production about the disease in the sheep producers' community, rather than prioritizing a more thorough diagnosis that could have potentially negative repercussions on their reputations as producers, but more positive consequences for biosecurity risks. These behaviors are understandable when we observe the reputational and economic loss that sheep producers can face and need to be framed within an international trend of neoliberal policies where governments have retreated from their responsibilities in biosecurity risks, relegating these responsibilities to farmers themselves (15). Paradoxically, the economic logic is eventually affected by the long-term effects of biosecurity risks.

These two factors combined, the complexity of the diagnosis and the potential and significant economic and reputational losses that are a consequence of the stigma associated with footrot disease, generate a state of anxiety for the sheep producer that may cloud his or her judgement coming up with an accurate diagnosis of the disease. According to sociologist Elias' (23) framework of involvement and detachment, this stigma can be conceptualized as a form of fantasy-like type of knowledge—excess of involvement—that is characteristic of states or situations where knowledge about something, in this case footrot disease, is insufficient to control it. The lack of knowledge about this disease among the producers' community is not only evident in our sample of open-ended responses, whose data we present in the results section below, but also has been found within the footrot literature, as reported above.

In order to offer a potential solution to the problem of institutional uncertainty and social stigma embedded in the manifestations of footrot disease, we provide three recommendations. First, we recommend a simplified message around the wording used for footrot to encourage more open dialogue to improve control of the disease. Second, we also recommend fostering a culture of trust (24) as a way to avoid concealing behaviors among producers, where sheep producers can collectively, rather than individually, solve the problem. Finally, we recommend that the burden of responsibility for biosecurity risks is shared by governments and sheep producers, and that governments make an effort to generate trust among the sheep producer community, working in collaboration with them rather than dictating them what to do or leaving them on their own coping with biosecurity risks that affect can the whole of society. Having institutions that do not elude their responsibility managing biosecurity risks and foster open communication could create an opportunity to collectively handle the disease, where institutions and producers collaborate to solve the problem.

## Methods

### Ethical Statement

All procedures performed in studies involving human participants were in accordance with the ethical standards of the La Trobe University SHE College Human Ethics Sub-Committee (SHE CHESC) under negligible risk project S16-93, as outlined by the National Statement on Ethical Conduct in Human Research (2007) and the Australian Code for the Responsible Conduct of Research (2007).

### Survey Distribution and Questions

Hardcopy surveys were distributed manually at two sheep producer events in 2016, in addition to being available online from June 2016 to August 2017 (Supplementary Data Sheet 1). Online responses were recorded using google forms and exported to Microsoft Excel for analysis. The survey consisted of 24 fixed and short answer questions, split into 3 sections; Part 1, property details; Part 2, current footrot diagnostics; and Part 3, improving footrot diagnostics. No individually identifying data was collected, and respondents had the option not to answer all questions or not to submit the survey. Questions were designed to investigate current understanding of footrot, and satisfaction of current footrot diagnostics, in addition to any thoughts the respondent had on future directions to improve disease management and control. Property detail questions were required to be completed before moving on to the next section, and included details about the postcode of property, average head of sheep, open or closed flock, primary breed and business. Part 2 included three short answer questions, two “check all that apply” questions, and two single selection options (including “unsure” as an option), and 1 Likert scale. Part 3 contained five short answer questions, two single selection options, including “unsure,” 1 Likert scale and three check box questions (Supplementary Data Sheet 1).

### Participants

Participants were convenience sampled volunteers, both in person and online. Advertising of the survey occurred on sheep or rural specific social media, online media, or in person at sheep specific events. Participants completed the survey of their own volition and at their leisure and could choose to give as much or as little information as they wished. In total, 45 responses from Australia were received.

### Analysis

Basic descriptive statistics were performed for the fixed answer questions using Microsoft Excel, to identify the most common responses and the response rates of individual questions. Answers for descriptive statistics were counted singularly. Results to all questions are not presented in the analysis due to the volume. A thematic analysis of the short answer responses was performed using grounded theory methods (25, 26) to identify common themes between participants, with multiple themes often identified in single answers. This was to provide an insight into general attitudes surrounding footrot. For this analysis, we combined general (25, 27) and constructivist (26, 28) principles of grounded theory, which conceptualize the search for emergent themes and coding phases of the analysis in two different ways: in the former case, the researcher is supposed not to have any pre-conceived knowledge of the topic (25); the latter one allows for the interpretation of the researcher to be part of the analysis [(26, 28), p. 228]. This latter constructivist approach suited the purposes of our analysis to evaluate sheep producers' understanding and handling of the disease, as detailed below. Some other more general principles of grounded theory methods such as theoretical sampling (26) or finding a main processual category (29) were not used here because they were irrelevant for our analysis, emphasizing more of a classification of main categories than a construction of theory.

This combination of approaches within the same method was reflected in how Principal author Nickala Best (NB) and Co-author Ramón Menéndez (RM) collaborated to do the analysis. RM performed the initial analysis as a layman on footrot disease. Although RM was familiar with grounded theory methods, he was not familiar with footrot disease. Using Nvivo software, RM conducted line-by-line and axial coding, coming up with a list of emerging themes in relation to the three main questions of the data: (1) the understanding of footrot as a disease; (2) diagnostics tools that sheep producers used to identify footrot disease in their affected flock; and (3) the control methods that they used to combat this disease. For the first question, RM and NB agreed NB's specific knowledge was needed to evaluate and classify sheep producers' understanding of the disease, so NB was called for the second stage of coding due to her more detailed understanding of footrot; she conducted a secondary coding based on her knowledge. This was done for the purposes of evaluating sheep producers' knowledge of the disease, which could not be assessed exclusively a from layman's (RM) perspective. After NB re-coding of emerging themes, some of the themes were reduced into broader categories based on specific knowledge of footrot—see the results section below for a description of these categories. For the second and third themes, RM's initial thematic analysis was maintained. Hence, a combination of layman (RM) and expert (NB) knowledge regarding footrot was used for thematic coding. Although the issue of inter-coding reliability has been considered as relatively important in the qualitative literature (30, 31), a criterion that was met in the first phase of our analysis, this is not so determining for the constructivist grounded theory approach that we have used in the second phase of the analysis.

## Results

### Farm Characteristics and Understanding of Footrot

A total of three hardcopy responses were received, 42 online responses from Australia, and one respondent from the USA, which was removed from analysis. In total 45 surveys, only from Australia, were used. Most of the respondents were from Victoria (60%), followed by Tasmania and New South Wales, with 17.7% each, and lastly South Australia with 4.3% (Table 1). No respondents were from Western Australia, Queensland, or the Northern Territory. Overall, the most common flock size of respondents was 1,001–5,000 head of sheep, with an open flock status producing meat. The flock status was considered “open” if new sheep, including rams, were introduced, while a “closed” flock was fully self-replacing.

**Table 1:** The property details, including size and business type, of respondents split by state.

		**Size (sheep numbers)**			**Flock status**		**Business**		
**State**	**Respondents (*n*)**	**0–1,000 (%)**	**1,001–5,000 (%)**	**5,001+ (%)**	**Open^a^ (%)**	**Closed^b^ (%)**	**Meat (%)**	**Wool (%)**	**Both (%)**
NSW	8	38	38	25	100	0	63	13	25
VIC	27	33	44	22	78	22	48	19	33
SA	2	0	100	0	50	50	100	0	0
TAS	8	13	25	63	63	100	13	25	63
Total	45	29	42	29	78	33	47	18	36

a *“open” flock defined—if new sheep, including rams, were introduced*.

b *“closed” flock defined as fully self-replacing*.

When asked to “Please briefly describe your understanding of footrot as a disease,” responses were categorized into different levels of understanding as described in Table 2. The responses received ranged from quite in depth, such as “A bacterial infection of the foot that is contagious and can be passed on to other sheep (in general) causes extreme lameness/smelling pussy feet/feet rotting off,—leading to lower productivity/if left untreated potentially death due to inability to walk” to very brief responses, for example, one respondent simply stated “poor.” Some of these answers represent part of the stigma associated with footrot disease as the sheep producer being “dirty” or unable to handle his or her farm and flock adequately. For example, this is the case in the two latter answers given: “smelling pussy feet/feet rotting off.” As mentioned, this is a stigma that we also found in the literature [(10), p. 369].

**Table 2:** A description of the criteria for each level of understanding when it comes to the footrot.

**Level**	**Description**	**Number of responses**
1	Identifies one symptom or cause correctly	17
2	Identifies two symptoms and/or causes and/or consequences correctly	8
3	Identifies all, symptoms, causes, and consequences correctly	7
NA	Answer is one or two words only, or understanding is not demonstrated in response	6

Most responses for understanding footrot were categorized as level 1, which is the poorest understanding (see Table 2), with roughly the same number of respondents giving level 2 and 3 answers, which are more complete understandings, with level three being the best. It does need to be noted that the length and depth of responses was purely up to the respondent, which may contribute to an over-representation of level 1 responses if care was not taken to fully answer the question.

### Current Footrot Diagnostics

Producers were asked if they had previously made a diagnosis of footrot, and if so, to indicate how it was made. There was a response rate of 97.8% (*n* = 44), with respondents able to select all the diagnostics that applied to them. From the 44 responses to this question, 20% selected three options, 23% chose two, and 57% chose a single option. Of the options, using oneself (self-diagnosis) was the most common for a diagnosis of footrot, followed by a veterinarian, the laboratory, not applicable and “other” (Table 3).

**Table 3:** The percentage of producers who have used various footrot diagnosis.

**Footrot diagnostic used**					
	**Veterinarian**	**Laboratory**	**Self**	**No answer**	**Other**
% Respondents	42	31	60	16	11

*Respondents were able to select more than one option*.

As a follow up to the previous question, the respondents were asked if they were satisfied with the diagnostics that were available to them. More people responded being “neither” satisfied or dissatisfied (27.9%, *n* = 12), with satisfied (20.9%, *n* = 9) and dissatisfied/very dissatisfied (18.6%, *n* = 8) the next most popular choices with very satisfied (14%, *n* = 6) being least popular response.

### Improving Footrot Diagnosis

Most respondents indicated that new diagnostic services would be best implemented in the paddock (93%), followed by the sale yards (35%) and regional surveillance (30%) (Table 4). The response rate was 95.5% (43/45).

**Table 4:** The percentage of respondents who indicated where they would like new diagnostic services to be implemented.

**Where do you think new diagnostic services would be best implemented?**					
	**Paddock**	**Saleyard**	**Regional surveillance**	**Other**	**No answer**
Percentage of respondents	93	35	30	0.00	5

*Respondents were able to choose more than one option*.

Following this question, we asked producers to elaborate on why they chose the responses they did. The most commonly detected themes were a fear of being labeled as having footrot, indicating the privacy of paddock testing was desirable (6 responses identified), and the speed of response for paddock diagnostics and getting to the root of the problem (6 responses identified).

There was a generally expressed concerned surrounding buying sheep with footrot. Some comments included: “we don't want to buy footrot, know if we got it and if our neighbors are hiding it,” “because current testing is not picking up sheep, especially those that are sold on auctions plus as “no known footrot history” when you know they are just avoiding the yards and should have to pass a test to be sold as footrot free.” Sentiments like these, where concern about tested pre-purchase or “picking it up” from sale yards, were expressed 4 times. Testing pre-sale was mentioned 4 times, with active monitoring by government mentioned a single time.

When asked about control programs, 80% of the respondents indicated they had implemented footrot control programs previously, and most considered the plan very successful (Table 5). Respondents were also asked to describe what treatments were used for the control programs, with footbathing identified 24 times, followed by foot inspections and paring (14), culling (12), and antibiotics (10). The sale of infected stock was also identified as a theme, occurring in responses as a control method seven times, and the isolation on-farm of infected sheep mentioned five times. In addition, using a combination of methods was explicitly mentioned three times. The use of professionals to help with control was only identified as a method four times.

**Table 5:** The percentage of respondents that thought their control or eradication plan of footroot was successful.

**Did you consider the control or eradication plan successful?**					
	**Very**	**Somewhat**	**Neither**	**Not very**	**No**
% Respondents	53	19	25	3	0

Roughly half of the respondents indicated that the improvement of diagnostics services would help them better control the disease, with 31% stating they are “unsure,” and ~9% stating “no” (Table 6).

**Table 6:** The percentage of respondents that thought improvement to diagnostic services would help, not help, were unsure, or chose not to answer.

**Would improving diagnostic services help to better control/manage footrot?**				
	**Yes**	**No**	**Unsure**	**No answer**
% Respondents	51	9	31	9

## Discussion

Most of the respondents were Victorian producers (27), as this was where most advertising for participants was carried out. All respondents provided details of their property, including production type (meat, wool, both), and if the flock was open or closed. Most respondents, 77.7%, stated their flocks were open. This creates an increased biosecurity risk, as footrot can be introduced through new stock, with this introduction identified as a concern by some producers. Victorian responses indicated that 48.1% had meat as the primary business, with this fitting with Victoria producing almost 50% of Australia's lamb. The results presented and discussed here are likely to have come from those producers who have an interest in footrot; typically, this means they will have had firsthand experience with the disease. This is useful for gaining insights into the actual experience of footrot but does present a potentially biased section of the population. The option to not answer questions, and to self-determine the level of detail given when answering short answer, means that some surveys were incomplete, or the answers given assumed the researcher would be able to extrapolate information, particularly for the understanding of footrot. In the instance of brief answers, no extrapolation was made, which may show an overrepresentation of poor understanding. The short-answer responses also mean that the themes identified are broad, however care has been taken to remove ambiguity from the themes identified.

The responses to “please briefly describe your understanding of footrot as a disease” showed a generally poor understanding of footrot (37.7%), which would inhibit efforts for control and eradication. As mentioned in the introduction, this poor understanding may reflect institutional uncertainty in the production of knowledge about this disease and be contributed by a desire to not recognize the disease, thus avoiding the stigma and rumors surrounding the disease within the farming community. These two factors combined, institutional uncertainty and the potential economic and reputational losses associated with the stigma, may cause the sheep producer to bias judgment (23). He or she is likely judging the situation favorably according to his or her most immediate economic benefits, downplaying clinical signs of the disease as “benign” when selling or communicating with other sheep producers.

As a result, the most commonly reported method of diagnosis was “self,” with 60% of respondents indicating they used themselves to identify footrot. Respondents were able to select more than one response here, and 20% chose three options, 23% choosing two and 57% choosing a single option; this indicates that more than one option was used in one instance, or multiple options have been used in multiple instances. Self-diagnosis conducted in private is therefore more subjective. The sheep producer may avoid consultation with experts because of lack of trust in institutions [(11), pp. 368–371] and the reputational and economic loss stigma involves. A veterinarian was used for a diagnosis by 42.2% of respondents, which is low when considering the expertise that can be utilized when engaging with a professional. Roughly equal numbers showed a moderate and good understanding of the disease (17.7, 15.5%), which is promising for control and eradication. The correct understanding is important for recognition of the disease, and subsequent actions to minimize and treat the condition.

The primary source of dissatisfaction and distrust appears to be in the current diagnosis. Approximately half of the respondents thought the improvement of diagnostic services would help to better control and manage footrot, with 31% “unsure,” with ~9% saying “no,” they do not think it would help, and ~9% not answering (Table 5). The improvement of diagnostic services would have to be in line with what was desired for use by producers (as above), while also addressing the social aspects of footrot to ensure maximum compliance and engagement with appropriate control measures.

The most frequent response for “where new diagnostic services should be implemented” was “paddock,” followed by “saleyard,” “regional surveillance,” and “no answer.” This could indicate a combination of reasons to deal with the disease mainly in the paddock, early detection and privacy. The preference for a paddock based diagnostic was identified to treat infection early—“Early detection quarantining, and treatment is the key to reducing risk of spread”—and get an indication of the overall picture on individual farms—“Paddock allows you to get a complete picture of the bacteria and its host.” These results may also indicate a non-manifested simultaneous desire of producers to keep diagnosis private, maintaining their own public reputation, while risking other reputations to provide their own peace of mind before bringing new stock in from a sale. The opportunity to use a diagnostics pre-purchase at the sale yards was the second desirable option, as identified as a theme by one third of respondents, linking to another identified theme of generally expressed distrust of other producers, “that [at the sale yards] is generally where we pick it up from.” The respondents apparently desire a level of guarantee that they are not bringing infection onto farm. With 77.78% of respondents indicating an open flock, this is a valid concern from a biosecurity view. On farm or in person diagnosis would guarantee valuable information about the diagnosis of others and try to avoid the situation of “buying footrot,” as mentioned by one of the respondents. This can help create trust by inspiring confidence in purchasing sheep and managing biosecurity at the same time, creating a sense of ownership of the results, as they are happening on property and in front of the producer. Generating an environment where this was routine, and results were given without judgement, should help to reduce some confusion and encourage open communication.

Satisfaction for control methods was somewhat, with the majority of sheep producers (53%) expressing success with treatment. As mentioned above, the variability of the disease, reflected in the gray distinction between benign and virulent types of it, can make this division harder for producers, and accommodate their judgement to their economic interests. That said, precisely because of the involvement of producers with their sheep and their hands-on experience, they can have some valuable insights into the nature of the disease and its successful control. This should be taken into account when experts work with producers. Most of their control approaches were conservative in use, using the traditional method of footbathing, but these could be enhanced—and the speed of recovery accelerated—if experts are consulted at the paddock to aid the recovery process.

Themes that were identified during this survey—selling sheep as a control option, distrust of neighbors, and a desire for secrecy—are detrimental to the control of footrot. Selling sheep spreads the disease, while being distrustful of neighbors and wanting to keep a diagnosis of footrot private means open communication for working together for control is difficult, with this response to understanding summarizing multiple themes identified: “Expensive. We caught virulent footrot from the neighbors 2 years ago and it was cheaper to destock than to try and treat”; “Treatment advice is mixed and success rates poor due mainly to biosecurity. Footrot in this area is widespread due to complacency and sheer lack of biosecurity.” Another response stands out for identifying the problem is not only a disease of sheep, but as a social issue: “We need to stop treating this disease like a social disease and the industry might recognize how wide-spread, particularly benign footrot.”

## Recommendations

Responses and themes like the ones mentioned above indicate that footrot is very complex both as a disease and socially. With the poor understanding of the disease, and lack of engagement with professionals for diagnosis and help, it's clear a different and more holistic approach is needed to change the social perceptions of the disease, before more cooperative control strategies can be approached. Based on the confusion surrounding footrot, both in producers understanding and diagnostics, a simplification of approach to clinical disease and diagnostic capability would be helpful. The removal of words such as “virulent” and “legislation” may help to remove some of the current distrust and stigma surrounding footrot. “Benign” footrot was discussed 6 times in short answer questions without prompting, identified as a concern by the respondents, yet the focus for control and legislation purely on virulent despite benign being of concern to producers. In a bid to end some confusion surrounding footrot, including factors such as sheep breed and environmental conditions that influence disease expression, the presence of asymptomatic carriers, and the desire not to be labeled as having virulent footrot, footrot could or should be considered as a single disease. “Footrot” would then simply encompass all disease symptoms, where diagnosis of infection could be based on a bacterial characteristic and presence.

However, broader changes are needed to produce a knowledge sharing environment where producers and institutions engage in accurate discussion about potential solution. For this to happen, it is necessary first to demystify the disease and break the link between it and its associated social stigma. To avoid this link, fostering a culture of trust that collectively solves the problem may prevent inaccurate diagnosis and encourage engagement with expert knowledge of the disease. According to sociologist Diego Gambetta's research on trust, the benefits of trust are significant for any type of society, “even in unpromising situations” [(24), p. 228]. Acting as if trust is important or as if there is a basis for trust constitutes the basis for building healthy communities. Societies that trust in spite of the odds are societies that on the whole tend to do better than others where trust is not fostered. One of the keys for trust is having open communication, which may significantly increase the chances of trusting behaviors succeeding. We acknowledge that fostering trust among the producers community or in institutions can be problematic [(10), p. 370, (15), p. 406], but trust is the key to collectively solve this problem.

Finally, the problems of trust identified within the farming community when managing footrot disease put in context of the neoliberal governmental policies that have affected biosecurity risks (15) and consequently producers' trust in institutions that deal with them [(11), pp. 361–363]. Therefore, the solution to this problem does not only refer to a change in vocabulary or a fostering of trust in relationships among producers, but also requires an acceptance of governments of their responsibilities for biosecurity risks. This does not imply that governments must produce a series of guidelines for sheep producers' communities to uncritically follow, but that institutions must make an effort to be transparent and approachable, generating a dialogue where producers and governments work together to find solutions for the disease. This might require additional funding to be invested in biosecurity risks and a different approach about communicating expert knowledge, but in any case, would not leave to economic logic the huge task of managing biosecurity risks. Diagnosis and control of footrot must be produced with expert help, but for that to happen trust on institutions and among producers themselves must happen first.

## Data Availability Statement

All datasets generated for this study are included in the article/Supplementary Material.

## Ethics Statement

The studies involving human participants were reviewed and approved by La Trobe University SHE College Human Ethics Sub-Committee. Written informed consent for participation was not required for this study in accordance with the national legislation and the institutional requirements.

## Author Contributions

NB, TB, GR, and BR: conceptualization. NB and RM: methodology, formal analysis, investigation, and writing—original draft preparation. NB, RM, GR, RS, BR, and TB: writing—review and editing. All authors contributed to the article and approved the submitted version.

## Conflict of Interest

The authors declare that the research was conducted in the absence of any commercial or financial relationships that could be construed as a potential conflict of interest.
