# Supplementary material for: The Consequences of Stigma for Knowledge Production: Sheep Producers' Attitudes to Footrot Diagnostics and Control in Australia
Source: Front Vet Sci. 2020 Jun 25;7:354. doi: 10.3389/fvets.2020.00354 (PMC7329981; doi:10.3389/fvets.2020.00354)
Supplement: Supplementary file 1 [file Data_Sheet_1.PDF]

Sheep Producers Attitudes Toward Current and New Footrot Diagnostics.

This survey is made up of fixed choice and open ended questions. It should take approximately 10 minutes to complete, and you are helping to contribute to improving our understanding of footrot! The survey is totally anonymous.

Section 2 is the Participant Information Statement, where you can find a description of the project and contact details, with Section 3, 4 and 5 the survey questions.

Your participation is very much appreciated!

Yours Sincerely,

Nickala Best, PhD Candidate

* Required

Skip to "Sheep Producers Attitudes Toward Current and New Footrot Diagnostics.."

Sheep Producers Attitudes Toward Current and New Footrot Diagnostics.

PARTICIPANT INFORMATION STATEMENT

Supervisor: Dr. Travis Beddoe, Senior Lecturer, Department of Animal, Plant and Soil Sciences, La Trobe University.
Email: T.Beddoe@latrobe.edu.au Phone: +61 9032 7400

PhD Candidate: Ms. Nickala Best, Doctor of Philosophy by Research, Department of Animal, Plant and Soil Science, La Trobe University.
Email: N.Best@latrobe.edu.au Phone: +61 9032 7439

Eligibility: Participants must be at least 18 years of age and able to fluently read and write English in order to fill out the survey independently. Participants must also own sheep.

Aim
We aim to investigate the attitudes of sheep producers and owners towards the current state of diagnostics available for footrot, and how new methods would be best implemented in order to improve control of the disease.

Project Benefits
This research is part of a larger project investigating improved methods for the diagnosis of footrot, by detecting the causative bacteria, Dichelobacter nodosus. We hope to determine from the survey the current attitudes of sheep producers and owners around the diagnostics presently available for footrot, and the level of satisfaction with these techniques. We also wish to know what diagnostic techniques producers would like ideally to have available, and how they perceive any new diagnostic tools would be best implemented for the improved control of the disease. This information will be used to inform the larger research scope to better serve industry.

What does the research involve?
Your only obligation is to allocate 10 to 30 minutes (maximum) to fill out the survey. The questions will be fixed choice and open ended questions which require you to detail descriptive factors about your personal experiences and opinions.

Inconvenience/discomfort
You shouldn’t experience any discomfort while answering the survey.

Identification/Security
No personal information will be collected, however a postcode is required. Surveys will be anonymous, and data stored on a password protected computer and Excel spreadsheet.

Withdrawal

You have the right to withdraw from active participation in this project at any time before submission of the survey. To withdraw do not submit a completed survey. Once surveys are submitted, withdrawal of data is not possible as the data collected is anonymous and your responses cannot be identified for removal. There are no disadvantages, penalties or adverse consequences for not participating or for withdrawing from the research.

Other information
Data may also be included in a thesis, presented at a conference and/or published in a scientific journal article, presented in a summary format with no individual identifiers.

Any general questions relating to this project should be directed to Ms. Nickala Best, of the La Trobe University Department of Animal, Plant and Soil Science, by email at N.Best@latrobe.edu.au or Phone: 03 9032 7439.

Any concerns or queries relating to how the project has been carried out should be directed to Dr. Travis Beddoe, either via Email: T.Beddoe@latrobe.edu.au or Phone: 03 9032 7400.

If you have any complaints or concerns about your participation in the study that the researcher has not been able to answer to your satisfaction, you may contact the Senior Human Ethics Officer, Ethics and Integrity, Research Office, La Trobe University, Victoria, 3086 (P: 03 9479 1443, E: humanethics@latrobe.edu.au). Please quote the application reference number S16-93.

By clicking next, you are agreeing that the data collected may be used as mentioned in 'other information'.

Skip to question 1.

Survey - Part 1
Property Details

1. **Post code of property** *

____

2. **Average head of sheep on property?** *

____

3. **Is it an open (including introduction of rams) or closed flock?** *
   Check all that apply.

   - [ ] Open
   - [ ] Closed

4. **What is your primary business (meat/wool etc)?** *

____

5. **What is your primary breed?** *

____

Skip to question 6.

Survey - Part 2
Current Footrot Diagnostics

6. **Please briefly summarise your understanding of footrot as a disease.**

____

____

____

____

____

7. **Have you diagnosed footrot previously using a vet, laboratory, self or other (please tick all that apply)?**

Check all that apply.

- [ ] Vet
- [ ] Laboratory
- [ ] Self
- [ ] Not applicable
- [ ] Other: ________________________________

8. **Was a diagnosis of virulent, benign or scald made (please tick all that apply)?**

Check all that apply.

- [ ] Virulent
- [ ] Benign
- [ ] Scald
- [ ] None
- [ ] Not applicable

9. **Do you consider 'scald' a separate disease to footrot?**

Mark only one oval.

- ( ) Yes
- ( ) No
- ( ) Unsure

10. **What footrot diagnostic services are available to you (please list)?**

____

____

____

____

____

11. **What is your preferred diagnostic method?**

____

____

____

____

____

12. **Are you satisfied with current footrot diagnostic services?**
Mark only one oval.

	1	2	3	4	5	
Very unsatisfied	⬭	⬭	⬭	⬭	⬭	Very satisfied

13. **Is cost a factor when deciding to diagnose footrot?**
Mark only one oval.

- ⬭ Yes
- ⬭ No
- ⬭ Unsure

Skip to question 14.

Survey - Part 3

Improving Footrot Diagnositcs

14. **What footrot diagnostic services would you like available (please tick all that apply, and if 'other', please state)?**
Check all that apply.

- ☐ Virulent and benign testing
- ☐ Serogrouping of the bacteria
- ☐ Predictive testing (identifying risk of lesions)
- ☐ Detection of bacteria in the soil and/or pasture
- ☐ None
- ☐ Other: ____________________

15. **How do you think improved footrot diagnostic services would affect your business (if unsure, please state 'unsure')?**

____
____
____
____
____

16. **Would you prefer to use a new diagnostic method or a current method if you suspected footrot in your flock?**
Check all that apply.

- ☐ New
- ☐ Current
- ☐ None
- ☐ Unsure

17. **Where do you think new diagnostic services would be best implemented?**
Check all that apply.

- [ ] Paddock
- [ ] Saleyard
- [ ] Regional surveillance (example: monitoring abattoirs)
- [ ] Other: ____________________

18. **Please briefly state why -**

____
____
____
____
____

19. **Have you implemented footrot control or eradication plans previously?**
Mark only one oval.

( ) Yes
( ) No

20. **If yes, please briefly describe the methods employed (please state NA if not applicable).**

____
____
____
____
____

21. **If yes, what method was used to diagnose footrot in that situation (please state NA if not applicable)?**

____

22. **Did you consider it successful (please leave blank if NA)?**
Mark only one oval.

	1	2	3	4	5	
No	( )	( )	( )	( )	( )	Very

23. **Would the improvement of diagnostic services help you to better control/manage footrot?**
Mark only one oval.

( ) Yes
( ) No
( ) Unsure

24. **Please provide a brief description of how (if you are unsure, please state 'unsure').**

Thank you!

Your time is greatly appreciated and has contributed to our research.

Powered by
Google Forms
